# Intraoperative prevention of postoperative hypoparathyroidism

**DOI:** 10.3389/fendo.2023.1206881

**Published:** 2023-11-08

**Authors:** Kristina Vabalayte, Anatoly Romanchishen, Aleksandra Somova

**Affiliations:** Federal State Budgetary Educational Institution of Higher Education “Saint-Petersburg State University”, St. Petersburg, Russia

**Keywords:** thyroidectomy, postoperative hypoparathyroidism, safe surgery, hypocalcemia, parathyroid hormone

## Abstract

**Objective:**

More than 30,000 thyroid surgeries are performed annually in the Russian Federation. The surgeries are relatively safe because of the prevention methods for postoperative complications. Currently, there is no single effective method of postoperative hypoparathyroidism prevention. This complication is frequently reported and may be health and life-threatening.

**Aim:**

We aimed to estimate the effectiveness of the intraoperative ICG-angiography and intrathyroid injection of Brilliant Green for the prevention of postoperative hypoparathyroidism.

**Material and methods:**

One hundred and forty-three thyroidectomies were performed. Patients were divided into three groups: intraoperative angiography was used in 24 cases; Brilliant Green was injected in 58 cases to identify parathyroid glands; the visual estimation of the parathyroid preservation was used in 61 cases. Calcium level was measured in all patients before and after surgery.

**Results:**

Calcium level in the serum before and after surgery was 2.37±0.14 and 2.27±0.17 in Group 1, 2.38±0.16 and 2.21±0.16 in Group 2, and 2.39±0.17 and 2.18±0.19 in Group 3. Postoperative hypocalcemia was more prominent in the group with the visually estimated PTG than in the two other groups. The differences in postoperative calcium levels in Groups 1 and 3 were statistically different. Pre- and postoperative Parathormone levels were 6.2±0.4 in Group 1, 5.6±0.57 in Group 2, and 3.5±0.32 in Group 3. Postoperative levels significantly differed in Groups 1 and 3 (p<0.01) and in Groups 2 and 3 (p<0.05).

**Conclusions:**

ICG-angiography and intrathyroid injection of the Brilliant Green are safe methods of identification and sparing of the parathyroid glands. The severity of hypocalcemia and hypoparathormonemia in Group 3 shows the necessity of finding new methods in endocrine surgery to improve patient outcomes.

## 1 Justification

Thyroid pathology has been a major medical problem among endocrinopathies for many years (1–5). Due to the COVID-19 pandemic, much fewer thyroid surgeries were performed in the Russian Federation in 2020 compared to 2019 (14,699 surgeries in 2019 vs 33,237 in 2020) (6), and surgical treatment is still necessary for many patients (2, 3). The use of neuromonitoring over the past years allows for the reduction of complications due to laryngeal nerve damage (7). The use of endovideo surgery allows for the improvement of a cosmetic outcome, which is important for the psychological comfort of the patient (8–10). A high risk of postsurgical hypoparathyroidism (PSH) remains a serious complication (3, 11–15) and cannot be underestimated. According to the British Association of Endocrine and Thyroid Surgeons, the incidence of transient and permanent PSH is 23.6% and 7.3%, respectively (16, 17). G. H. Sakorafas et al. reported that the major cause of PSH is accidental parathyroidectomy, which is observed in 17.7% of cases (18). S. Martin et al. and J. J. Diez report an even higher incidence of PSH of 31% and 48.3%, respectively (14, 19).

Multiple techniques to reduce the risk of PSH have been suggested in the history of endocrine surgery. Many surgeons still believe that the best method of evaluation of the preservation of the parathyroid glands (PTG) is their visual control. Others disagreed pointing out a possible blood vessel damage of PTG during their inspection, and there are also doubts about the clear criteria of such an evaluation (18, 20). PTG autotransplantation has been recommended in cases of accidental parathyroidectomy or PTG vessel damage; however, it is not always possible to assess the preservation of PTG. Many authors argue against this procedure (15, 20) and are in doubt about the integrity of vascular perfusion of PTG. Some reports indicate that ligation of the lower thyroid artery may affect PTG blood supply (16). The use of special magnifying optics allows for the reduction of the risk of PSH in these cases (21).

The use of different dyes to identify PTG has become another method to improve the safety of thyroid surgery. The most popular of them are Methylene Blue (MB) and Aminolevulinic acid (ALA). The use of MB was initially proposed by N. E. Dudley in 1971 (22). But only in 2018, S. L. Hillary reported his work where he estimated the fluorescent ability of this substance injected intravenously (23). A.V. Zubkov suggested injecting the MB into the lower thyroid artery during surgery and got excellent results in experiments proving the effectiveness of MB (24). Although used by many, MB is not safe and could lead to toxic encephalopathy (25–28). A similar problem exists for the ALA; although a good visualization of PTG after the use of ALA (29, 30) was reported, it was associated with phototoxicity in half of the patients (25, 28).

All the issues mentioned above prove that currently there is no single reliable method of preventing PSH. Even in cases of transient PSH, it is associated with longer hospital stays, additional lab tests, and the use of additional medicines, i.e., it involves additional costs. Moreover, PSH may be life-threatening for the patients; therefore, it should be avoided as incompatible with the concept of safe surgery.

## 2 The aim of the study

The purpose of this study is to evaluate the efficiency of intraoperative angiography with Indocyanine Green (ICG-AG) and intrathyroid injection of 1% Brilliant Green (BG) water solution for PSH prevention.

## 3 Materials and methods

### 3.1 The place of the study

A. M. Nikiforov Federal Government Centre of Urgent and Radiology Medicine and St. Petersburg Mariinsky City Hospital.

### 3.2 Time of the study

The study was performed between 2018 and 2021.

### 3.3 The studied patients

#### 3.3.1 Exclusion criteria

The patient’s refusal to participate in the study.

#### 3.3.2 Inclusion criteria

First time thyroid surgery.

#### 3.3.3 Indication for surgery

Follicular thyroid tumor (Bethesda IV), thyroid carcinomas (Bethesda VI), toxic thyroid goiter, and autoimmune thyroiditis with compression of neck and/or mediastinal organs.

### 3.4 The study design

The design description:

- single center,
- experimental,
- dynamic (calcium and parathormone (PH) levels were estimated in all patients before and on Day 1 after surgery),
- prospective (follow-up time: up to 14 days),
- double selective,
- no placebo control group,
- non-randomized.

### 3.5 The description of medical intervention (for interventional studies)

**Figure 1:**
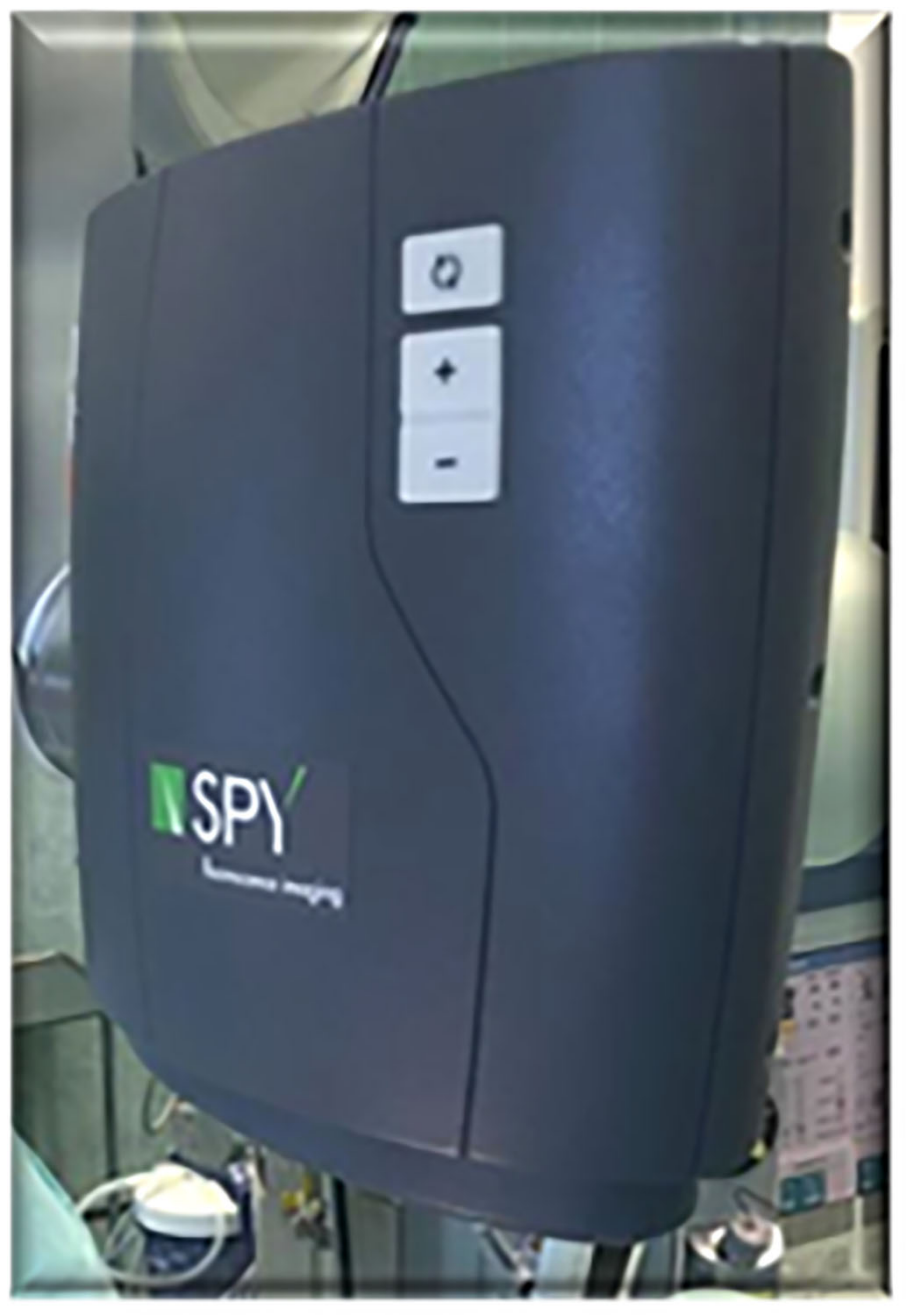
The SPY visualisation system.

**Figure 2:**
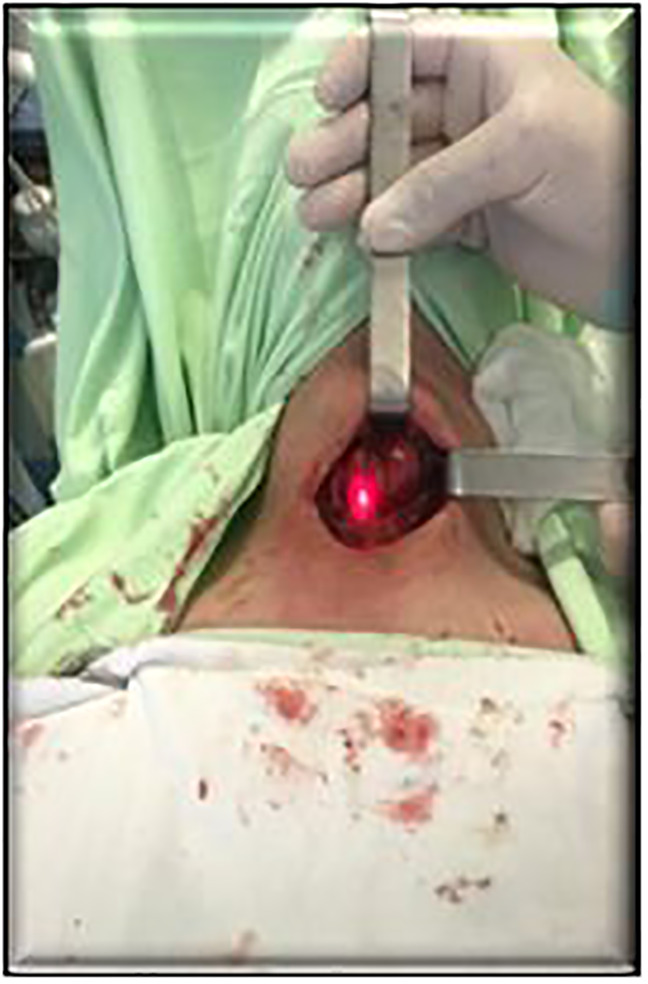
Installing the system before angiography procedure.

The SPY visualization system (**Figure 1**) was used for intraoperative angiography including the source of the near-infrared spectrum, which causes fluorescence of ICG. The system was installed before the angiography procedure at the focal distance of 30 cm above the surgical wound (**Figure 2**). The contrast was injected twice: before thyroidectomy to identify PTG and after the surgery for PTG perfusion assessment. The dose of ICG injected i.v. did not exceed 5 ml (the average dose was approximately 8.5 mg per person). The visual fluorescence was detected at 20-30 sec after injection, with the peak registered at 50 sec. The time for identification of PTG did not exceed 4 min.

The fluorescence data were evaluated by a scale from 0 to 2, where the ICG 0, 1, and 2 corresponded to no, weak, or good signal, respectively.

**Figure 3:**
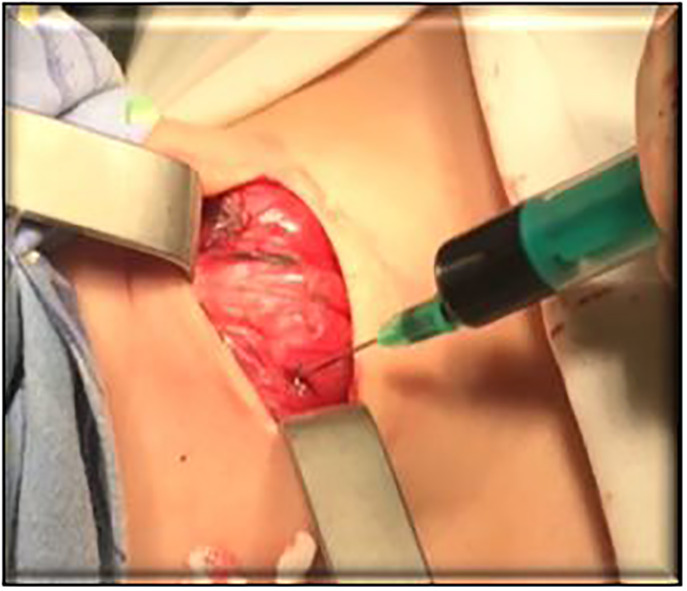
Performing an injection of B Green.

**Figure 4:**
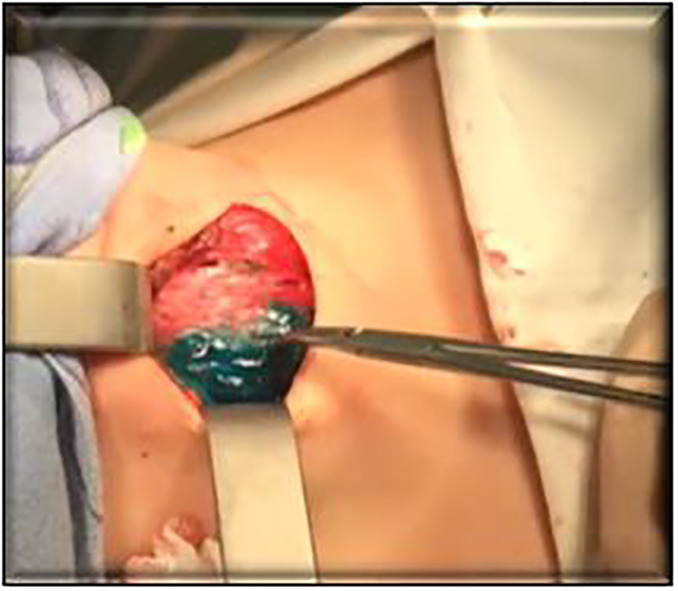
Staining of thyroid tissue.

The injection of B Green water solution was performed right before the determination of the surgical borders (**Figures 3**, **4**). The surgery was performed after intrathyroid injection of BG along the tissue border staining. Thus, only the thyroid lobe where the contrast was injected was stained green, while PTG was stained negative. It simplified the visualization of PTG and its preservation.

In all three groups, calcium level was evaluated on Day 1 after surgery.

### 3.6 Methods

#### 3.6.1 Approval of ethical committee

All patients provided informed consent for participation in this study. The study was approved by the ethical committees of both hospitals based on the Helsinki Declaration of 1964.

#### 3.6.2 Inclusion criteria assessment

Data of pre-surgical examination.

#### 3.6.3 Additional methods

Thyroid ultrasound using the expert class instruments, fine needle aspiration biopsy of thyroid nodes with subsequent Bethesda scale for evaluation of samples.

#### 3.6.4 Laboratory tests

Calcium, PH, free T3 and T4, and TSH. Biochemical Analyser AU 5800 (Beckman Coulter, USA) and the Dia Liaison XL (Italy) were used for the measurement of calcium and PH levels, respectively.

SPY Visualisation Systems, NerveMonitor System (InoMed, Germany) for laryngeal nerve function control, Univet binocular lenses (x2.5), and Ethicon Harmonic Scalpel were also used during the surgery.

### 3.7 Statistical analysis

Calcium level before and after surgery was compared in all patients using The MS Soft Excel 2010 software. The statistical significance was estimated with the Student t-test and p<0.05 was considered significant.

## 4 Results

A total of 147 patients (11 men and 136 women) between the age of 28 and 72 (mean age 58.3 ± 4.2). Patients included in the study had various pathologies (diffuse toxic goiter, thyroid cancer, and multinodular goiter).

The patients were divided into three groups:

1. Group 1 (28 patients) where ICG-AG was used.
2. Group 2 (58 patients) where intrathyroid injection of BG was used.
3. Group 3 (61 patients) where only visual assessment of PTG was used.

Before the surgery, calcium level in all three groups was comparable: 2.37 ± 0.14, 2.38 ± 0.16, and 2.39 ± 0.17 mmol/l in Groups 1, 2, and 3, respectively (reference ranges for calcium: 2.10-2.55 mmol/l). After surgery, hypocalcemia was observed in all three groups and was 8%, 14%, and 26% in the group with intraoperative angiography, BG use, and visual evaluation of the PTG preservation, respectively. The mean calcium level after the surgery was 2.27± 0.17, 2.21± 0.16, and 2.18 ± 0.19 mmol/l in Groups 1, 2, and 3, respectively, where the difference between Groups 1 and 3 was statistically significant (p < 0.05).

PH levels before the surgery were also not significantly different (8.6 ± 0.56, 8.0 ± 0.43, 8.2 ± 0.41 in Groups 1, 2, and 3, respectively (reference ranges for PH 2.0-9.3 pmol/l)). The mean PH level after the surgery was 6.2±0.4 in Group 1, 5.6±0.57 in Group 2, and 3.5±0.32 in Group 3. The PH levels in Groups 1 and 3 were significantly different. The PSH was observed in 1%, 14%, and 22%. There were no accidental parathyroidectomy in groups with ICG-AG and BG use according to histology results. There were no adverse effects in the groups with ICG-AG and BG i.v. injection.

It was not always easy to identify PTG from surrounding tissues after the first injection of ICG; however, after the second contrast injection (after thyroidectomy), identification of PTG was not problematic. This allowed for the selection of patients who were affected by PTG vascularization after surgery, and these patients could receive more attention during the follow-up. In none of the patients, PTG was graded as zero and PSH was transient in this group.

There were no problems with PTG visualization in Group 2. The use of BG proved to be reliable in the prevention of accidental parathyroidectomy, which allows for more care with PTG during surgery and avoids accidental PTG removal. However, this technique does not allow for the assessment of the initial PTG and its blood supply integrity after the removal of thyroid tissue in cases of repeated surgeries. The risk of neurovascular PTG damage still exists despite an improved identification of PTG.

## 5 Discussion

Our study shows the value of ICG-AG and intrathyroid BG injection for the prevention of PSH in all patients after thyroidectomy.

The first report that described the intraoperative use of ICG-AG in thyroid surgeries was published in 1914 by Y. J. Suh et al. The author suggested a method of identifying the PTG based on animal studies. There were some difficulties in PTG identification because the contrast was accumulated by all tissues with good vascularization, and identification of the PTG was not clearly reliable. However, the study demonstrated good fluorescence of the contrast and the safety of this method (3). Later studies have demonstrated that the contrast dose indicated in the seminal study was insufficient (4).

Recently R. Parfentiev et al. published a study where they compared the visual estimation of the PTG and intraoperative angiography. The calcium level was evaluated in all patients on days 1 and 7-15 after the surgery. The postsurgical PSH level was 17.86% and 6.67% in the group with the visual estimation of the PTG and in the angiography group, respectively. In all patients, the complication was transient. The study suggested that this method was safe and effective (31).

Yu et al. in their work evaluated the ICG-AG in robotic thyroid surgeries from bilateral axillo-breast approach access and reported that this method was safe, convenient, and efficient for the identification and preservation of PTG (4).

Maser, C. et al. used ICG-AG to study PTG identification in cases of PTG pathology and found that there is stronger fluorescence in PTG pathology than in normal PTG. This suggested that the method is valuable not only in thyroid but also in parathyroid surgeries. No difficulties in PTG identification have been reported (32).

Several studies reported ICG-AG not only for PTG localization during surgery but also to reveal the patients at risk of PSH after thyroidectomy. The contrast was injected after the main stage of the surgery thyroid removal had been done and the PTG perfusion was estimated. In all the studies, the patients at risk of PSH were revealed. No adverse events have been reported (11, 33).

Rare (0.00167%) adverse events reported so far were connected with allergic reactions to iodine. This type of adverse event should be taken into consideration before the surgery, as it is a contraindication to ICG use (34).

Our data as well as the data in previous literature suggested that ICG-AG use can reduce the incidence of PSH, reveal the patients at high risk of the condition, shorten hospital stay in patients with preserved PTG blood supply, and avoid PTG autotransplantation during surgery and calcium administration after surgery.

Thus, ICG-AG is a promising method for the identification and preservation of the PTG which needs further development. However, since the groups in our study are small, and the cost of equipment is high, it is reasonable to consider alternative methods of PTG visualization. One such method is intra-thyroid injection of BG. The contrast spreads diffusely in the thyroid tissue, while the PTG remains contrast-free (35). This method can be competitive with the more expensive and difficult-to-perform ICG-AG.

### 5.1 The clinical significance of the results

The high incidence of PSH cannot be underestimated. The methods described in the study could reduce the risks of this complication and reveal patients who need a more careful postsurgical follow-up.

### 5.2 The limitations of the method

The weakness of the intraoperative angiography is the high cost of the equipment.

## 6 Conclusions

The intraoperative ICG-AG may be considered a reasonable and safe method of identification and preservation of the PTG. This method allows for the discovery of patients at risk of PSH.

BG injection looks relatively inferior compared to ICG-AG; more cases of PSH were observed when BG was used. However, this method improves intraoperative identification of PTG and costs much less than angiography.

The high degree of postsurgical hypocalcemia in Group 3 points to the fact that not only visual identification and assessment of PTG viability are important but also the need for novel advanced technology.

It has been suggested that “The best way to find the PTG during surgery – is to find the right surgeon” (31). However, looking for new ways to solve the problem makes this world better. The professional perfection of the surgeon is definitely of the highest importance, but even the best of them can make mistakes, which is illustrated in the statistics of PSH described above. The development of new advanced methods will allow us to better protect our patients from additional risks of PSH.

## 7 Further studies

New studies with larger patient groups and detailed analysis of predictors of PSH are advisable.

## Data availability statement

The raw data supporting the conclusions of this article will be made available by the authors, without undue reservation.

## Ethics statement

Ethical approval was not required for the studies involving humans because in this case, only voluntary medical consent was required for the operation. The studies were conducted in accordance with the local legislation and institutional requirements. The participants provided their written informed consent to participate in this study.

## Author contributions

AS: conception of the study, analysis and interpretation of the data, and writing the manuscript. KV: conception and design of the study, obtaining the data, and adding to the manuscript. AR: conception of the study, proofreading, and approval of the final manuscript. All authors contributed to the article and approved the submitted version.
